# Bioactive Compounds from *Tithonia diversifolia* Aerial Parts Against Eggs and Infective Larvae of the Parasitic Nematode *Haemonchus contortus*

**DOI:** 10.3390/pathogens14090884

**Published:** 2025-09-04

**Authors:** Jorge Alberto Cortes-Morales, Agustín Olmedo-Juárez, Victoria Michelle Tapia-Molina, Manases González-Cortazar, Alejandro Zamilpa, Pedro Mendoza-de Gives, Abel Villa-Mancera, Bernardo Sachman-Ruiz, Filiberto Anzures Olvera

**Affiliations:** 1 Laboratorio de Fitoquímica y Productos Naturales del Centro de Investigación en Biodiversidad y Conservación, Universidad Autónoma del Estado de Morelos, Av. Universidad 1001, Col. Chamilpa, Cuernavaca C.P. 62209, Mexico; ing_cortesmorales@yahoo.com.mx; 2 Centro Nacional de Investigación Disciplinaria en Salud Animal e Inocuidad (CENID SAI-INIFAP), Carretera Federal Cuernavaca-Cuautla No. 8534/Col. Progreso.Jiutepec, A.P. 206-CIVAC, Jiutepec C.P. 62574, Mexico; pedromdgives@yahoo.com (P.M.-d.G.); bsachman@gmail.com (B.S.-R.); 3 Ingeniería en Biotecnología, Universidad Politécnica del Estado de Morelos, Cuauhnáhuac 566, Lomas del Texcal, Jiutepec C.P. 62574, Mexico; victoriatapia1873@gmail.com; 4 Centro de Investigación Biomédica del Sur, Instituto Mexicano del Seguro Social, Argentina No. 1, Xochitepec C.P. 62790, Mexico; gmanases2000@gmail.com (M.G.-C.); azamilpa_2000@yahoo.com.mx (A.Z.); 5 Facultad de Medicina Veterinaria y Zootecnia, Benemérita Universidad Autónoma de Puebla, Tecamachalco C.P. 75460, Mexico; abel.villa@gmail.com; 6 Centro de Investigación Regional Pacífico-Sur (CIRPAS-INIFAP), Iguala de la Independencia C.P. 40000, Mexico; faov225@hotmail.com

**Keywords:** forage plants, Asteraceae, hydroxycinnamic acid, gastrointestinal nematodes, small ruminants

## Abstract

Small ruminant production under grazing conditions plays a crucial role in the global primary sector economy. However, these animals are highly susceptible to gastrointestinal nematodes (GINs), which significantly impact their health and welfare. Given the increasing resistance to conventional anthelmintics, there is a pressing need to explore sustainable alternatives, such as plant secondary metabolites. This study aimed to identify phenolic compounds with anthelmintic activity from *Tithonia diversifolia* aerial parts, using *Haemonchus contortus* as a biological model. Egg hatching inhibition (EHI) and larval mortality assays were used to evaluate the anthelmintic activity of a hydroalcoholic extract (HA-E), an aqueous (Aq-F) and ethyl acetate fraction (EtOAc-F), and eight bioactive subfractions (TdR1-TdR8) obtained from EtOAc-F. The identification of major compounds was performed using HPLC-PDA. The E-HA and EtOAc-F achieved 100% EHI at 40 and 4 mg/mL, respectively. The subfractions TdR2 (EC_90_ = 0.55 mg/mL), TdR3 (EC_90_ = 0.12 mg/mL), and TdR4 (EC_90_ = 0.26 mg/mL) exhibited the highest ovicidal activity. In the larval mortality test, EtOAc-F showed an LC_85_ of 56.74 mg/mL. The major identified compounds included cinnamates, hydroxycinnamic acids (e.g., caffeic acid and chlorogenic acid), gallates, flavonoids (flavones and flavanones), and coumarins. These findings support the potential of *T. diversifolia* as a promising natural source for the control of GINs in small ruminants.

## 1. Introduction

The growing resistance of gastrointestinal nematodes (GINs) to anthelmintic drugs represents one of the primary challenges in sheep and goat grazing systems, with significant implications for both public and veterinary health. Among these parasites, *Haemonchus contortus* is the most prevalent worldwide. This hematophagous nematode consumes approximately 0.05 mL of blood per day, leading to anaemia, weight loss, reduced productivity, and, in severe cases, sudden death in infected animals [1,2]. Diseases caused by GINs, such as *H. contortus*, result in considerable economic losses in meat and milk production, as well as their derived products [3].

The conventional treatment of GIN infections relies on synthetic anthelmintics, including benzimidazoles, imidazothiazoles, macrocyclic lactones, and salicylanilides. However, the improper and indiscriminate use of these drugs has led to the emergence of resistant GIN strains, thereby reducing the efficacy of these treatments [4,5,6]. Consequently, alternative strategies have been explored to reduce the dependence on conventional anthelmintics. These include pasture rotation, the selection of genetically resistant animals, vaccines, biological control, and the use of plants rich in secondary metabolites that possess health benefits for animals [7,8,9].

*Tithonia diversifolia*, commonly known as “botón de oro” or “árbol maravilla”, is a plant of the Asteraceae family native to Central and South America. In Mexico, it is widely distributed in states such as Chiapas, Guerrero, Oaxaca, and Veracruz [10]. Traditionally, this species has been used in ethnomedicine to treat ailments like diabetes, diarrhoea, fever, stomach pain, muscle pain, bruises, and infections [11]. Pharmacological evaluations of the extracts from the different parts of *T. diversifolia* have demonstrated multiple biological activities, including anti-inflammatory, hypoglycaemic, analgesic, antimicrobial, hepatoprotective, antioxidant, anticancer, insecticidal, antimalarial, and anthelmintic effects [12,13,14].

Due to its high nutritional value, *T. diversifolia* has been used as a feed for poultry, fish, goats, sheep, and cattle. Some studies have proposed its use and ideal shrub silvopastoral systems in tropical environments aimed at sustainable ruminant production or as a forage bank. These reports have mentioned that *T. diversifolia* is a viable alternative for daily livestock feeding due to its high protein content, favourable mineral content, low fibre content, and potential to reduce greenhouse gas emissions such as methane, which contributes to enhancing the production and quality of meat and milk [15,16,17,18].

The phytochemical composition of *T. diversifolia* has been extensively studied, with over a hundred secondary metabolites isolated and identified from different extracts. These include a wide variety of sesquiterpenes, diterpenes, flavonoids, hydroxycinnamic acids, coumarins, and essential oils [16,19]. Although several studies have confirmed the anthelmintic activity of *T. diversifolia* extracts against *H. contortus*, there is a lack of bioguided research in order to identify the specific secondary metabolites for this activity. Therefore, the objective of this study was to identify the secondary compounds with anthelmintic activity present in a hydroalcoholic extract from *T. diversifolia* aerial parts, using *H. contortus* as a biological model.

## 2. Materials and Methods

### 2.1. Plant Material

*Tithonia diversifolia* aerial parts (stems and leaves) were collected from an experimental crop established at the Iguala-INIFAP Experimental Field in Guerrero, Mexico. The plant material was dried at room temperature under shade for three weeks and then ground into particles of approximately 3–5 mm in size. The ground material was stored in a cool and dry place until extraction.

### 2.2. Obtaining the Hydroalcoholic Extract (HA-E)

Ground plant material (800 g) was macerated with 8000 mL of a hydroalcoholic solution (30% methanol–70% distilled water) for 24 h in darkness. The hydroalcoholic extract (HA-E) was then filtered through cotton, gauze, and filter paper and concentrated under reduced pressure in a rotary evaporator (R-300, Büchi, Flawil, Switzerland) at 50 °C, 90 rpm, and 140 mbar until the methanol was eliminated. The aqueous phase of the HA-E (5000 mL) was stored at 4 °C until further biological evaluation and chemical fractionation.

### 2.3. Liquid–Liquid Separation of HA-E

The aqueous phase of HA-E (1000 mL) was subjected to a liquid–liquid portioning process using ethyl acetate (EtOAc) in a 1:1 ratio in a separation funnel. This procedure was repeated five times, processing a total of 5000 mL of HA-E. The bipartition resulted in an aqueous fraction (Aq-F) and an organic fraction (EtOAc-F), which were concentrated using a rotary evaporator, brought to dryness via lyophilisation and stored at 4 °C until further biological evaluation and chemical fractionation.

### 2.4. Chemical Fractionation of EtOAc-F

The EtOAc-F (5000 mg) was subjected to a chemical separation process using open column chromatography (40 cm × 5 cm) previously packed with 30 g of silica gel (normal phase, 70–230 mesh, Merck, Darmstadt, Germany). Elution was carried out using a hexane–acetone gradient system, beginning with a 100:0 ratio and gradually increasing the polarity by 5% acetone increments until reaching a 50:50 hexane–acetone ratio. A total of 105 fractions (15 mL each) were obtained, which were analysed via thin-layer chromatography (TLC) and grouped into eight subfractions based on their chemical similarity. All samples of these chromatographic process were analysed via TLC on silica gel 60 F254 (Merck, Germany) under UV light at 254 and 360 nm. The natural product polyethylene glycol reagent (NP-PEG; 1% methanol solution of diphenylboryloxyethylamine, followed by 5% ethanol polyethylene glycol) was used as a chemical detection reagent [20].

### 2.5. Compound Identification Through HPLC

The identification of secondary compounds in the HA-E and fractions was performed using an HPLC system (Delta Prep 4000, Waters, Milford, MA, USA) equipped with a Waters 2695 separation module, a photodiode array detector (Waters 996), and Pro Empower software (Waters). For chemical separation, a reverse-phase Supelcosil LC-F column (250 mm × 4 mm and 5 μm particle size) (Merck, Darmstadt, Germany) connected to a guard column was used. The elution was carried out using a mobile phase consisting of 0.5% TFA solution (solvent A) and acetonitrile (solvent B). The gradient system was as follows: 0–1 min, 0% B; 2–3 min, 5% B, 4–20 min, 30% B; 21–23 min, 50% B; 24–25 min, 80% B; 26–27 min, 100% B; and 28–30 min, 0% B. The sample injection volume was 10 μL, and the flow rate was maintained at 0.9 mL min 1 [21]. Absorbance was measured at 270, 330, and 360 nm to identify phenolic acids, flavonoids, and coumarin derivatives.

### 2.6. Obtaining Biological Material

The *H. contortus* IVM-susceptible (*Hc*IVM-S) strain was used for this study. This strain was originally obtained from naturally infected lambs at “Las Margaritas Ranch” in Hueytamalco village, Puebla State, Mexico, and has since been maintained under cryopreservation conditions. Nucleotide sequence data for this strain are available in the GenBank^TM^ database (BioProject number: PRJNA877658) [6,22].

Eggs and infective larvae (L3) were obtained from faeces collected from an egg-donor sheep (21 kg of bodyweight) previously infected with 4900 *H. contortus* L3 larvae. The animal was treated according to the Mexican Official Standard for care/welfare (NOM-062-ZOO-1999), was housed in an individual cage and was fed with alfalfa hay, commercial concentrate food, and water ad libitum. The sheep received food equivalent to 3% of their body weight twice a day.

### 2.7. Haemonchus contortus Eggs Recovery

Egg recovery was performed using sequential sieves according to the technique described by Coles et al. [23] with minor modifications. The faecal sample (30–40 g) was macerated and homogenised with tap water. The faecal solution (300 mL) was distributed into twelve Falcon tubes (25 mL each) and filled to 50 mL with saturated saline solution (42%); then, the tubes were centrifuged at 3500 rpm for 5 min. The supernatant was sieved and washed with tap water through a 75 and 32 μm mesh to collect the eggs.

### 2.8. Haemonchus contortus Infective Larvae (L3) Recovery

To recover *H. contortus* L3 larvae, faeces were collected in a basin for 24 h from the egg-donor sheep. To ensure adequate oxygenation and an optimal hatching of larvae, the faeces were macerated with clean tap water and mixed with foam rubber in a basin until 90% humidity. The faecal cultures were covered with aluminium foil and incubated for seven days at a temperature between 25 and 31 °C. The L3 larvae were extracted from faecal cultures using the Baermann funnel technique and cleaned through gravity sedimentation and density gradient centrifugation using distilled water and filter paper. Subsequently, the L3 were unsheathed via exposure to a 0.187% sodium hypochlorite solution for eight minutes with constant agitation; they were immediately rinsed with distilled water, and serial dilutions were made to obtain a stock larvae solution containing 100 ± 15 larvae/50 μL for larval mortality bioassay [24].

### 2.9. Egg Hatch Inhibition (EHI) Assay

The ovicidal activity of HA-E and its fractions was evaluated using the EHI assay in 96-well microtiter plates. Bioassays were performed in triplicate, with four repetitions for each treatment and its proper controls (*n* = 12). An aqueous suspension (50 μL) containing approximately 100 ± 15 eggs was deposited in each well; then, 50 μL of each extract, fraction, or proper control was deposited in each well to reach a total volume of 100 μL. The treatments were evaluated as follows: HA-E at 5, 10, 20 and 40 mg/mL, the Aq-F at 2.5, 5, and 10 mg/mL, the EtOAc-F at 0.5, 1, 2, and 4 mg/mL, and the subfractions obtained from EtOAc-F (TdR2, TdR3, TdR4, TdR5, TdR6 and TdR7) at 0.062, 0.125, 0.25, 0.5, 1, 2 mg/mL. Distilled water and 3% methanol were evaluated as negative controls, and as a positive control, thiabendazole (0.1 mg/mL, dissolved in DMSO) was used [25]. To estimate the EHI, counts of eggs and hatched larvae from each well were performed under an optical microscope at 4×. The EHI percentage for each treatment and respective control was determined by applying the following formula:

$$\%EHI=\;\frac{Number\;of\;eggs\;}{Number\;of\;eggs+number\;of\;larvae}\times 100$$

### 2.10. Larval Mortality Assay

The larval mortality evaluation of the EtOAc-F and its fractions was carried out in 96-well microtiter plates. Bioassays were performed in triplicate, considering four repetitions for each treatment and its proper controls (n = 12). The treatments were EtOAc-F at 3.75, 7.5, 15, 30 and 60 mg/mL and the subfractions at 10, 20, 30 and 40 mg/mL. Distilled water, 3% methanol, and a solution of Polyvinylpyrrolidone (PVP) at 60 mg/mL were evaluated as negative controls, and ivermectin (IVM) at 5 mg/mL was used as a positive control. In each well of the titration microplate, 50 µL of each treatment and respective controls and 50 µL of aqueous suspension containing 100 ± 15 *H. contortus* L3 larvae were added. The plates were incubated at room temperature (25–30 °C) for 72 h. To read the bioassay, 10 aliquots of 10 µL were taken from each well, and live and dead larvae were counted under a 4× optical microscope. The percentage of larval mortality was determined using the following formula:

$$\%Larval\;mortality=\;\frac{Number\;of\;dead\;larvae\;}{Number\;of\;living\;larvae+Number\;of\;dead\;larvae}\times 100$$

### 2.11. Statistical Analysis

The data obtained in the egg-hatching inhibition assay and the percentage of larval mortality were subjected to an analysis of variance (ANOVA), under a completely randomised design using the general linear model of the SAS program 9.4 version. A Tukey test was performed to identify differences between the means of each treatment. Treatments showing a concentration-dependent effect were subjected to regression analysis using the PROC PROBIT program to determine the effective concentrations (EC), 50 and 90, and the lethal concentrations (LC), 50 and 85.

## 3. Results

### 3.1. Hydroalcoholic Extract, Fractions, and Subfractions Yields

Maceration of 800 g of the *T. diversifolia* aerial parts produced a 12.8% (102.4 g) yield of the HA-E. The bipartition of the HA-E produced a yield of 93.65% of Aq-F and 6.35% of EtOAc-F. The subfractions TdR1 to TdR8 produced the following yields: TdR1 = 0.31% (0.0167 g), TdR2 = 1.75% (0.0944 g), TdR3 = 3.13% (0.1689 g), TdR4 = 16.91% (0.9117 g), TdR5 = 53.70% (2.8943 g), TdR6 = 6.16% (0.3324 g), TdR7 = 2.98% (0.1609 g), and TdR8 = 2.30% (0.6868 g).

### 3.2. Chemical Identification of Tithonia diversifolia HA-E and Fractions

**Figure 1:**
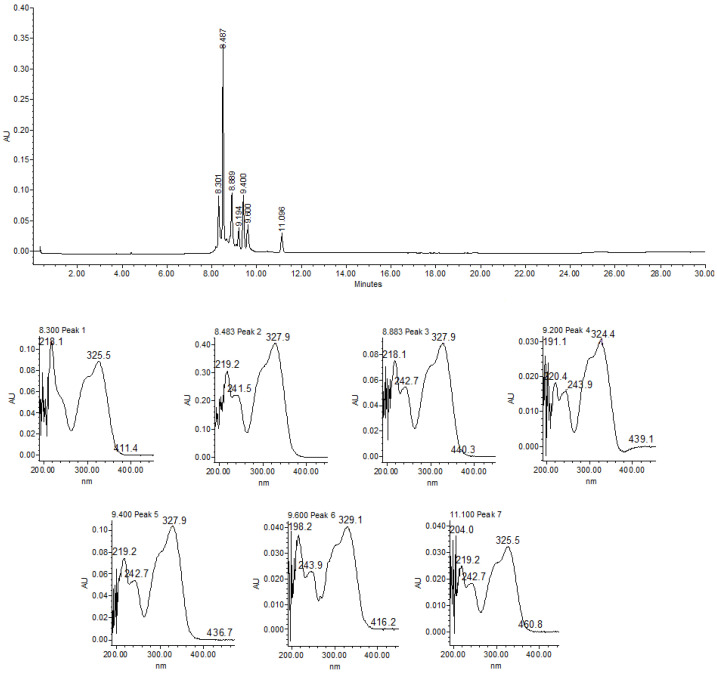
Chromatogram of Tithonia diversifolia of hydroalcoholic extract (HA-E) obtained via HPLC-PDA and recorded at λ = 300 nm. AU = Absorbance units.

Figure 1 shows the chromatogram of the *T. diversifolia* hydroalcoholic extract (HA-E) obtained via HPLC-PDA. The UV absorption spectra analysis and retention time (Rt) of the major peaks present in HA-E revealed the presence of chlorogenic acid (Rt = 8.3 min; λnm = 241.4 and 325.5), caffeic acid (Rt = 8.48 min; λnm = 241.5 and 327.9), and derivatives of these two compounds. The Aq-F exhibited the same chemical profile as HA-E (Figure S1).

**Figure 2:**
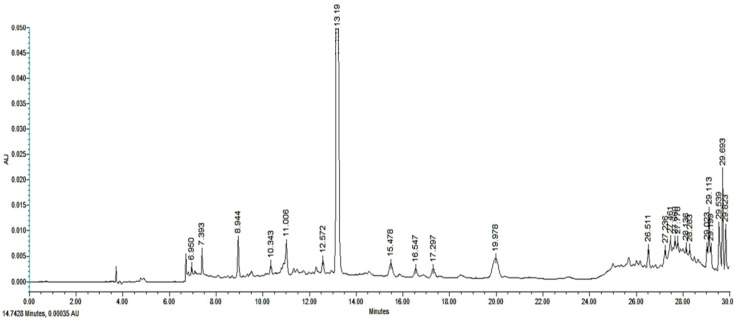
Chromatogram of ethyl acetate fraction (EtOAc-F) from *Tithonia diversifolia* obtained via HPLC-PDA and recorded at λ = 300 nm. AU = Absorbance units.

The EtOAc-F chromatogram exhibited a major peak with a UV absorption spectrum typical of a caffeic acid derivative (Rt = 13.19); however, it showed 24 minor peaks with UV absorption spectra typical of hydroxybenzoic acids, cinnamic acid derivatives, caffeic acid derivatives, and coumarins (Figure 2).

After the chemical fractionation of AcOEt-F, eight fractions (TdR1-TdR8) were obtained, of which only six (TdR2-TdR7) showed anthelmintic activity and were analysed via HPLC-PDA. The chromatogram of the TdR2 fraction exhibited four major peaks, compounds that were identified as low-polarity coumarins according to their Rt and UV absorption spectrum (Figure S2). The chromatogram of the TdR3 revealed eighteen peaks, corresponding to coumarins of medium and low polarity, a hydroxybenzoic acid, a coumaric acid derivative, a caffeic acid derivative, and five cinnamic acid derivatives (Figure S3). The chromatogram of the TdR4 fraction exhibited nine peaks, among which one flavanone, five coumarins, two caffeic acid derivatives, and one low-polarity cinnamic acid derivative were identified (Figure S4). The chromatogram of the TdR5 fraction exhibited eight peaks, corresponding to six coumarins, one hydroxybenzoic acid and one caffeic acid derivative were identified (Figure S5). The chromatogram of the TdR6 fraction exhibited fourteen peaks, among which twelve coumarins, one caffeic acid derivative, and one flavone were identified (Figure S6). The chromatogram of the TdR7 fraction revealed the presence of nine coumarins, and two caffeic acid derivatives were identified (Figure S7).

### 3.3. EHI of Tithonia diversifolia HA-E and Fractions

The HA-E showed an EHI of 100% at 40 mg/mL, similar to the result obtained with the positive control (Table 1). The fractionation of HA-E yielded an Aq-F and an EtOAc-F, which showed EHI values of 86.5% at 10 mg/mL and 99.5% at 2 mg/mL, respectively. These results were statistically similar to HA-E.

Table 2 shows the EHI results of the subfractions (TdR2-TdR7) obtained from *T. diversifolia* EtOAc-F. Subfractions TdR2, TdR3, and TdR4 exhibited statistically similar activity, each achieving more than 96% EHI at 1 mg/mL. Meanwhile, subfractions TdR5, TdR6, and TdR7 showed significantly lower effectiveness, with EHI values of 46.9, 84.1, and 75% at 1 mg/mL. All subfractions showed a concentration-dependent hatching inhibition.

Table 3 shows the effective concentrations (EC_50_ and EC_90_) of HA-E and its fractions required to inhibit 50% and 90% of *H. contortus* egg hatching. The HA-E exhibited an EC_90_ of 18.51 mg/mL, whereas Aq-F and EtOAc-F showed EC_90_ of 10.69 and 1.39 mg/mL, respectively. The most bioactive subfractions were TdR2, TdR3, and TdR4 with EC_90_ values of 0.553, 0.123, and 0.282 mg/mL, respectively.

### 3.4. Larval Mortality of Tithonia diversifolia EtOAc-F and Its Subfractions

Since HA-E and Aq-F did not exhibit larvicidal activity against *H. contortus*, Table 4 presents only the percentage of larval mortality caused by EtOAc-F at different concentrations. The EtOAc-F induced 84.6% mortality at 60 mg/mL, decreasing in a concentration-dependent manner to 44% at 3.7 mg/mL. Regression analysis of the larval mortality data indicated that the lethal concentration required to cause 50 and 85% mortality was 4.76 and 56.74 mg/mL, respectively.

## 4. Discussion

Research on specialised metabolites obtained from plants with anthelmintic potential has gained significant attention worldwide as a sustainable alternative for controlling gastrointestinal nematodes in livestock and reducing the use of synthetic drugs. In this context, *Tithonia diversifolia* is a forage plant with high nutritional value and high anthelmintic potential. The results of this study indicate that HA-E, Aq-F, and EtOAc-F obtained from the aerial parts of *T. diversifolia* contain metabolites such as chlorogenic acid, caffeic acid, hydroxybenzoic acids, cinnamic acid derivatives, and coumarins that exhibit ovicidal and larvicidal activity. The HA-E showed 100% EHI at 40 mg/mL, and Aq-F and EtOAc-F were 4 and 20 times more effective than HA-E, respectively, in the EHI assay. Additionally, EtOAc-F caused over 80% larval mortality at 60 mg/mL. To date, few studies have investigated the anthelmintic activity of *T. diversifolia* against *Haemonchus contortus*. Duarte et al. [26] evaluated the inhibitory effect of *T. diversifolia* leaf and stem powder on *H. contortus* larval development in sheep faecal cultures, reporting reductions of 31% and 64.9% for stem and leaf powder, respectively, at 326.6 mg/g of faecal cultures. Buyi et al. [27] evaluated a *T. diversifolia* ethanolic extract on the motility inhibition of *H. contortus* adults obtained from goats at different concentrations. The authors report motility inhibition and 100% mortality of *H. contortus* at 2.5 h of exposure to the *T. diversifolia* ethanolic extract at 20 mg/mL. A phytochemical analysis of that extract revealed the presence of tannins, saponins, alkaloids, coumarins, and flavonoids.

To our knowledge, this is the first study to conduct a bioguided investigation identifying chemical compounds from *T. diversifolia* with anthelmintic activity against *H. contortus*.

Among the eight subfractions obtained from the EtOAc-F chemical fractionation, TdR2, TdR3, and TdR4 stood out due to their high effectiveness, achieving 100% EHI at 2 mg/mL. Notably, TdR3 subfraction showed the most potent activity, with aEC_50_ and EC_90_ values of 0.028 and 0.123 mg/mL, respectively, making it 11.30 times more effective than the EC90 (1.39 mg/mL) of EtOAc-F.

In the larval mortality assay, the activity observed in EtOAc-F was not retained at the same magnitude in its subfractions (TdR4, TdR5, and TdR7), suggesting that its anthelmintic effect could result from a synergistic or additive interaction among compounds. However, in the present study, combinations of subfractions were not evaluated, and therefore, the potential additive or synergistic effects remain to be determined. These subfractions contained high-polarity coumarins, cinnamic acid derivatives, caffeic acid derivatives, and hydroxybenzoic acids—although in lower concentrations and different proportions compared to the original EtOAc-F. These compound types have been isolated from other plant Asteraceae species and have shown significant ovicidal and larvicidal activity. In line with our findings, Jasso-Díaz et al. [28] evaluated a *Tagetes filifolia* methanolic extract against *H. contortus* eggs and larvae and reported 99% EHI at 20 mg/mL and 89% larval mortality at 200 mg/mL. In that study, chlorogenic acid was identified in the fraction that showed the greatest ovicidal activity. Cortes-Morales et al. [29] performed a bioguided chemical study using a methanolic extract from *Baccharis conferta* aerial parts against *H. contortus* eggs and found that the EtOAc-F achieved 100% EHI at 2 mg/mL, and a subfraction obtained from EtOAc fractionation, containing a dicaffeoylquinic acid, showed an EHI greater than 90% at 1 mg/mL.

In the present study, coumarins were identified as major compounds in all bioactive subfractions. Although most research on coumarins has focused on plant-parasitic nematodes, von Son-de Fernex et al. [30] demonstrated the ovicidal activity of the coumarin 2H-chromen-2-one isolated from *Gliricidia sepium* (Fabaceae) against *Cooperia punctata*, which inhibited hatching and embryonic development at 1.1 mg/mL. Escareño-Díaz et al. [31] also tested the coumarin 2H-chromen-2-one on the egg hatching and larval exsheathment inhibition of *C. punctata*, demonstrating that its EC_50_ decreased from 0.74 mg/mL to 0.23 and 0.17 mg/mL when combined with the flavonols quercetin and rutin, respectively. Cortes-Morales et al. [32] obtained coumarin-rich fractions with ovicidal activity against a mixed strain of gastrointestinal nematodes resistant to albendazole sulfoxide, ivermectin, and levamisole from an EtOAc fraction obtained from *Brongniartia montalvoana* aerial parts.

Several studies have documented the anthelmintic activity of cinnamic and hydroxybenzoic acid derivatives present in other plant species against gastrointestinal nematodes of small ruminants. For instance, cinnamic acid, ferulic acid, *p*-coumaric acid, caffeic acid, and their derivatives, identified in species such as *Acacia cochliacantha*, *Senegalia gaumeri*, *Pterogyne nitens*, *Pithecellobium dulce*, and *Chamaecrista nictitans*, have demonstrated important anthelmintic activity against *H. contortus* [21,33,34,35,36]. Hydroxybenzoic acids have also been identified in plant extracts and fractions with anthelmintic activity against *H. contortus*. For instance, gallic acid and protocatechuic acid are two hydroxybenzoic acids that have shown efficacy against gastrointestinal nematodes in small ruminants and other species [37,38,39,40,41].

Consistent with our findings, Becerril-Gil et al. [42] demonstrated that fractions obtained from *Arceuthobium vaginatum* ethyl acetate extract inhibited the hatching of *H. contortus eggs*. A phytochemical analysis revealed the presence of cinnamic acid derivatives, coumarins, and hydroxybenzoic acids such as protocatechuic acid.

Future studies should explore the combinations of hydroxycinnamic acids (e.g., caffeic acid and chlorogenic acid) with hydroxybenzoic acids (e.g., gallic acid and protocatechuic acid) in order to determine potential synergistic effects and identify the most effective bioactive interactions.

## 5. Conclusions

These findings support the potential of *T. diversifolia* as a promising natural source for the control of GIN in small ruminants. A chemical analysis of the ethyl acetate fraction revealed that the secondary metabolite groups most likely responsible for the anthelmintic activity are coumarin, hydroxybenzoic acids, and hydroxycinnamic acids. Further studies are needed to characterise the identity of the chemical compounds present in *T. diversifolia* that show anthelmintic activity.

## Figures and Tables

**Table 1:** Egg hatching inhibition percentage (%EHI) of *Tithonia diversifolia* hydroalcoholic extract (HA-E) and its aqueous (Aq-F) and ethyl acetate (EtOAc-F) fractions against *Haemonchus contortus* after a 48 h incubation period.

Treatments	Mean of Eggs and Larvae Recovered		%EHI ± s.d
	Eggs	Larvae	
Distilled water	3.08	126.16	2.43 ± 0.59 ^d^
Methanol (3%)	4.25	127.25	3.19 ± 2.13 ^d^
Thiabendazole (0.1 mg/mL)	129.91	0	100.00 ^a^
HA-E (mg/mL)			
40.0	111.91	0	100.00 ^a^
20.0	99.50	15.50	86.69 ± 23.04 ^ab^
10.0	100.75	24.50	81.12 ± 26.70 ^ab^
5.0	41.75	80.25	34.39 ± 5.81 ^cd^
Aq-F (mg/mL)			
10.0	99.25	15.00	86.57 ± 7.19 ^ab^
5.0	33.87	76.62	31.56 ± 8.30 ^cd^
2.5	3.83	115.25	3.16 ± 1.14 ^d^
EtOAc-F (mg/mL)			
4.0	128.83	0.00	100.00 ^a^
2.0	124.08	0.50	99.54 ± 0.78 ^ab^
1.0	74.67	41.00	62.49 ± 28.00 ^cd^
0.5	16.50	97.50	14.46 ± 12.02 ^d^
Variation coefficient			21.12
R^2^			0.94

^a–d^ Means with different letters in the same column represent statistically significant differences *p* < 0.05. s.d = standard deviation, n = 12.

**Table 2:** Egg hatching inhibition percentage (%EHI) of subfractions (TdR2-TdR7) obtained from *Tithonia diversifolia* ethyl acetate fraction against *Haemonchus contortus* after a 48 h incubation period.

Treatments	Mean of Eggs and Larvae Recovered		%EHI ± s.d
	Eggs	Larvae	
Distilled water	3	126.75	2.40 ± 1.28 ^mn^
Methanol (3%)	4.75	127	3.67 ± 1.59 ^mn^
Thiabendazole (0.1 mg/mL)	132.25	0	100.00 ^a^
TdR2 (mg/mL)			
2.0	123	0	100.00 ^a^
1.0	123.75	4.5	96.55 ± 1.18 ^abc^
0.5	122.25	19.75	86. 14 ± 1.00 ^de^
0.25	96.75	32.75	74.80 ± 4.28 ^fg^
0.125	48	55.5	46.67 ± 7.03 ^h^
TdR3 (mg/mL)			
2.0	147.75	0	100.00 ^a^
1.0	146.5	0	100.00 ^a^
0.5	138	1	99.28 ± 0.59 ^ab^
0.25	134.75	3.25	97.62 ± 1.13 ^abc^
0.125	130	15.75	89. 33 ± 1.94 ^bcd^
0.062	98.75	29.5	76.56 ± 4.77 ^efg^
TdR4 (mg/mL)			
2.0	136.75	0	100.00 ^a^
1.0	96.75	1	98.97 ± 1.19 ^ab^
0.5	81	2.5	97.12 ± 1.74 ^ab^
0.25	97.5	13	88.61 ± 3.50 ^cd^
0.125	66.5	33.25	66.79 ± 10.40 ^g^
0.062	21.5	99	17.96 ± 1.41 ^jkl^
TdR5 (mg/mL)			
2.0	135.25	28.25	82.88 ± 5.18 ^def^
1.0	75.5	86.5	46.90 ± 5.74 ^h^
0.5	30	156.75	16.11 ± 4.10 ^jkl^
0.25	18.75	159.75	10.49 ± 2.31 ^klmn^
TdR6 (mg/mL)			
2.0	115.25	2.25	98.13 ± 1.84 ^abc^
1.0	101.25	19.5	84.10 ± 3.39 ^def^
0.5	43.25	84.4	34.06 ± 4.35 ^i^
0.25	30.75	118.75	20.57 ± 2.52 ^jk^
TdR7 (mg/mL)			
2.0	136	16.75	89.03 ± 6.46 ^bcd^
1.0	119	39.75	75.02 ± 5.43 ^fg^
0.5	32.5	109.25	22.55 ± 7.10 ^j^
0.25	20.25	129.75	13.57 ± 2.59 ^jklm^
Variation coefficient			0.99
R^2^			6.36

^a–n^ Means with different letters in the same column represent statistically significant differences, *p* < 0.05. s.d = standard deviation, n = 12.

**Table 3:** Effective concentrations required to inhibit 50 and 90% (EC_50_ and EC_90_) of *Haemonchus contortus* eggs hatching after 48 h of exposure to *Tithonia diversifolia* HA-E and its fractions.

Treatments	EC_50_ (mg/mL)	Confidence Interval		EC_90_ (mg/mL)	Confidence Interval		Prediction Equations
		Lower Limit	Upper Limit		Lower Limit	Upper Limit	
HA-E	6.80	6.41	7.49	18.51	16.83	20.69	y = 0.3439ln(x) − 0.1596
Aq-F	6.36	6.02	6.69	10.69	10.00	11.57	y = 0.6626ln(x) − 0.7263
EtOAc-F	0.84	0.80	0.88	1.39	1.39	1.50	y = 0.6807ln(x) + 0.616
TdR2	0.139	0.125	0.154	0.553	0.505	0.612	y = 0.2502ln(x) + 0.992
TdR3	0.028	0.022	0.034	0.123	0.112	0.136	y = 0.2348ln(x) + 1.3346
TdR4	0.111	0.104	0.117	0.282	0.260	0.308	y = 0.3689ln(x) + 1.3109
TdR5	1.074	1.016	1.132	2.771	2.515	3.121	y = 0.3629ln(x) + 0.474
TdR6	0.560	0.531	0.589	1.349	1.251	1.469	y = 0.3912ln(x) + 0.7267
TdR7	0.783	0.700	0.777	1.915	1.775	2.086	y = 0.361ln(x) + 0.6093

**Table 4:** Larval mortality percentage of the EtOAc-F obtained from HA-E of *Tithonia diversifolia* aerial parts against *Haemonchus contortus* infective larvae after a 72 h incubation period.

Treatments	Mean of Larvae Recovered		%Mortality ± s.d
	Dead	Alive	
Distilled water	0.7	60.7	1.27 ± 1.60 ^e^
Methanol (3%)	0.3	70.7	0.53 ± 1.03 ^e^
PVP (60 mg/mL)	2.2	72.0	2.83 ± 2.44 ^e^
Ivermectin (5 mg/mL)	73.3	12.7	100 ^a^
EtOAc-F (mg/mL)			
60	76.8	14.0	84.61 ± 4.13 ^a^
30	73.3	19.7	78.35 ± 6.87 ^ab^
15	65.5	27.8	69.72 ± 9.97 ^bc^
7.50	55.1	37.7	58.64 ± 10.47 ^c^
3.75	41.1	49.8	44.11 ± 9.62 ^d^
Variation coefficient			12.25
R^2^			0.96

^a–e^ Means with different letters in the same column represent statistically significant differences, *p* < 0.05. s.d = standard deviation, n = 12.

## Data Availability

Data are contained within the article.

## Supplementary Materials

The following supporting information can be downloaded at https://www.mdpi.com/article/10.3390/pathogens14090884/s1, Figure S1: Chromatogram and UV absorption spectra of the *Tithonia diversifolia* aqueous fraction (F-Aq) obtained via HPLC-PDA at λ = 330 nm; Figure S2: Chromatogram and UV absorption spectra of the *Tithonia diversifolia* TdR2 subfraction obtained via HPLC-PDA at λ = 330 nm; Figure S3: Chromatogram and UV absorption spectra of the *Tithonia diversifolia* TdR3 subfraction obtained via HPLC-PDA at λ = 330 nm; Figure S4: Chromatogram and UV absorption spectra of the *Tithonia diversifolia* TdR4 subfraction obtained via HPLC-PDA at λ = 330 nm; Figure S5: Chromatogram and UV absorption spectra of the *Tithonia diversifolia* TdR5 subfraction obtained via HPLC-PDA at λ = 330 nm; Figure S6: Chromatogram and UV absorption spectra of the *Tithonia diversifolia* TdR6 subfraction obtained via HPLC-PDA at λ = 330 nm; Figure S7: Chromatogram and UV absorption spectra of the *Tithonia diversifolia* TdR7 subfraction obtained via HPLC-PDA at λ = 330 nm; Figure S8: Chromatogram and UV absorption spectra of the commercial standard chlorogenic acid, λ = 330 nm; Figure S9: Chromatogram and UV absorption spectra of the commercial standard caffeic acid, λ = 330 nm.
