# Supplementary material for: Bioactive Compounds from Tithonia diversifolia Aerial Parts Against Eggs and Infective Larvae of the Parasitic Nematode Haemonchus contortus
Source: Pathogens. 2025 Sep 4;14(9):884. doi: 10.3390/pathogens14090884 (PMC12472740; doi:10.3390/pathogens14090884)
Supplement: Supplementary file 1 [file pathogens-14-00884-s001.zip › pathogens-3835858-supplementary.pdf]

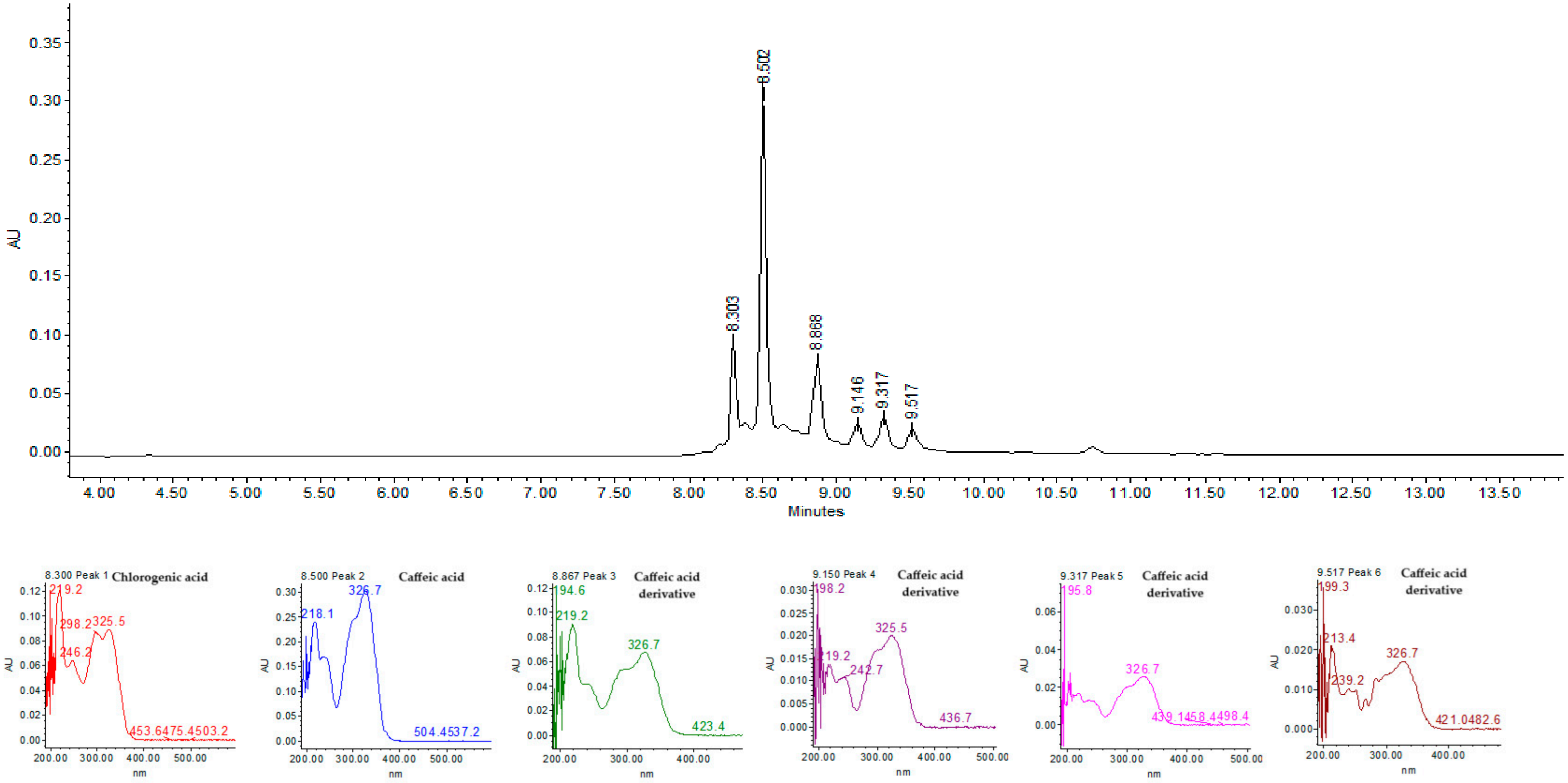


**Figure S1**. Chromatogram and UV absorption spectra of the *Tithonia diversifolia* aqueous fraction (F-Aq) obtained by HPLC-PDA at $\lambda$=330 nm

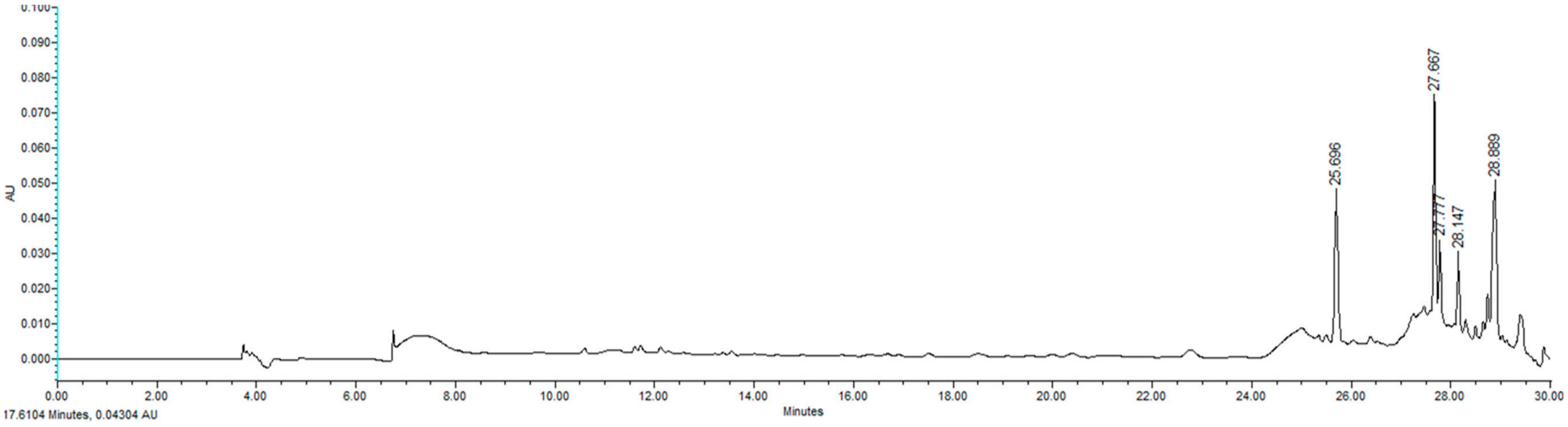


**Coumarin derivatives**

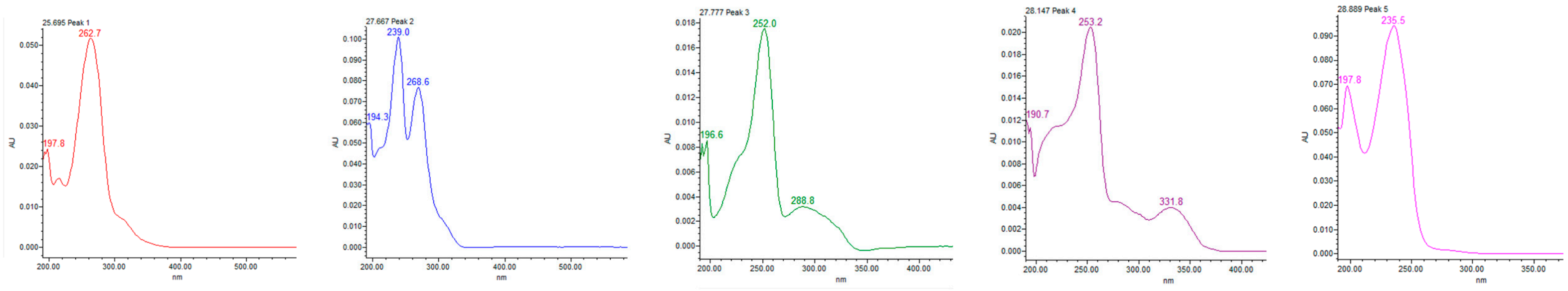


**Figure S2.** Chromatogram and UV absorption spectra of the *Tithonia diversifolia* TdR2 subfraction obtained by HPLC-PDA at $\lambda$=330 nm

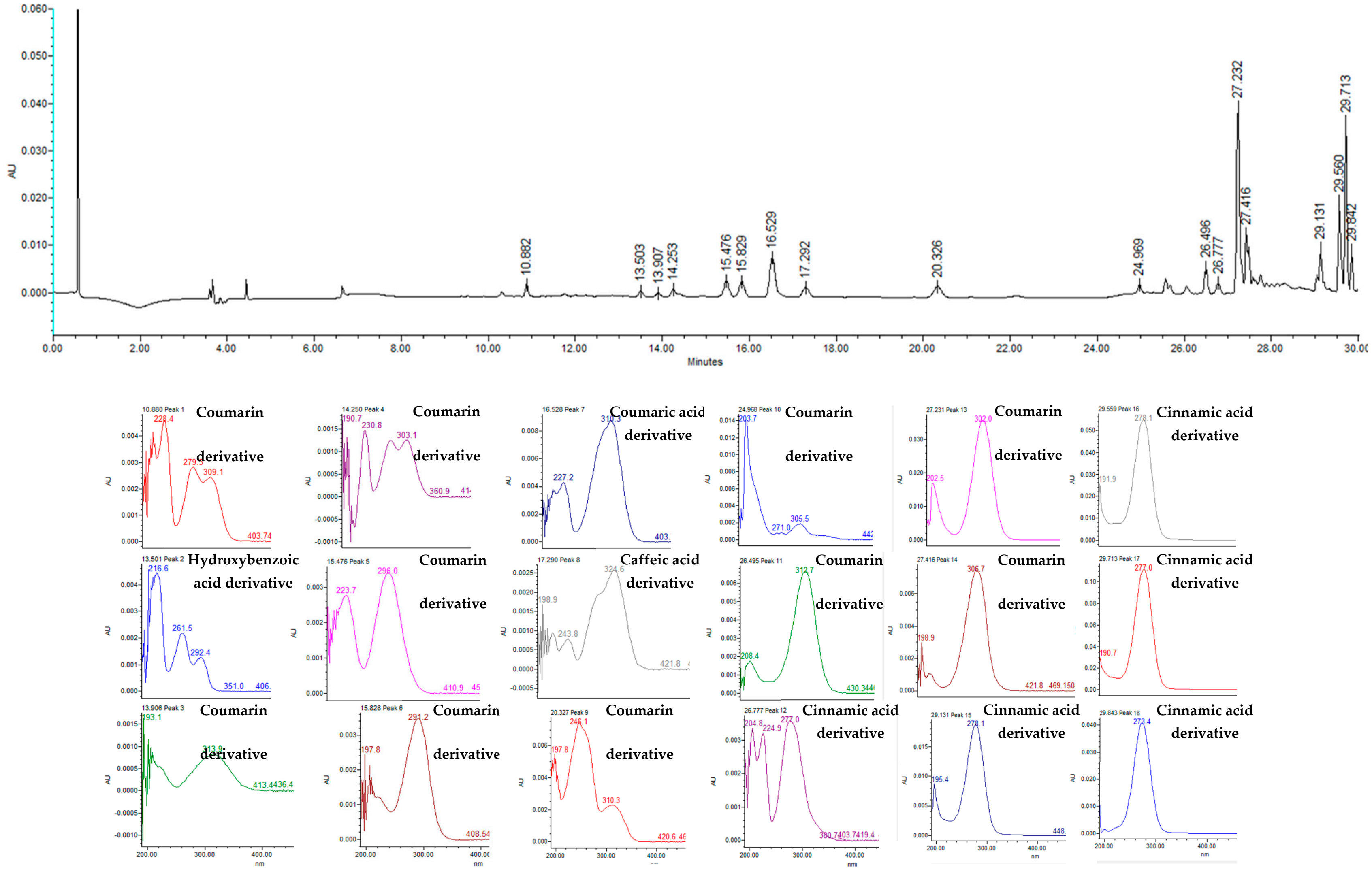


**Figure S3.** Chromatogram and UV absorption spectra of the *Tithonia diversifolia* TdR3 subfraction obtained by HPLC-PDA at $\lambda$=330 nm

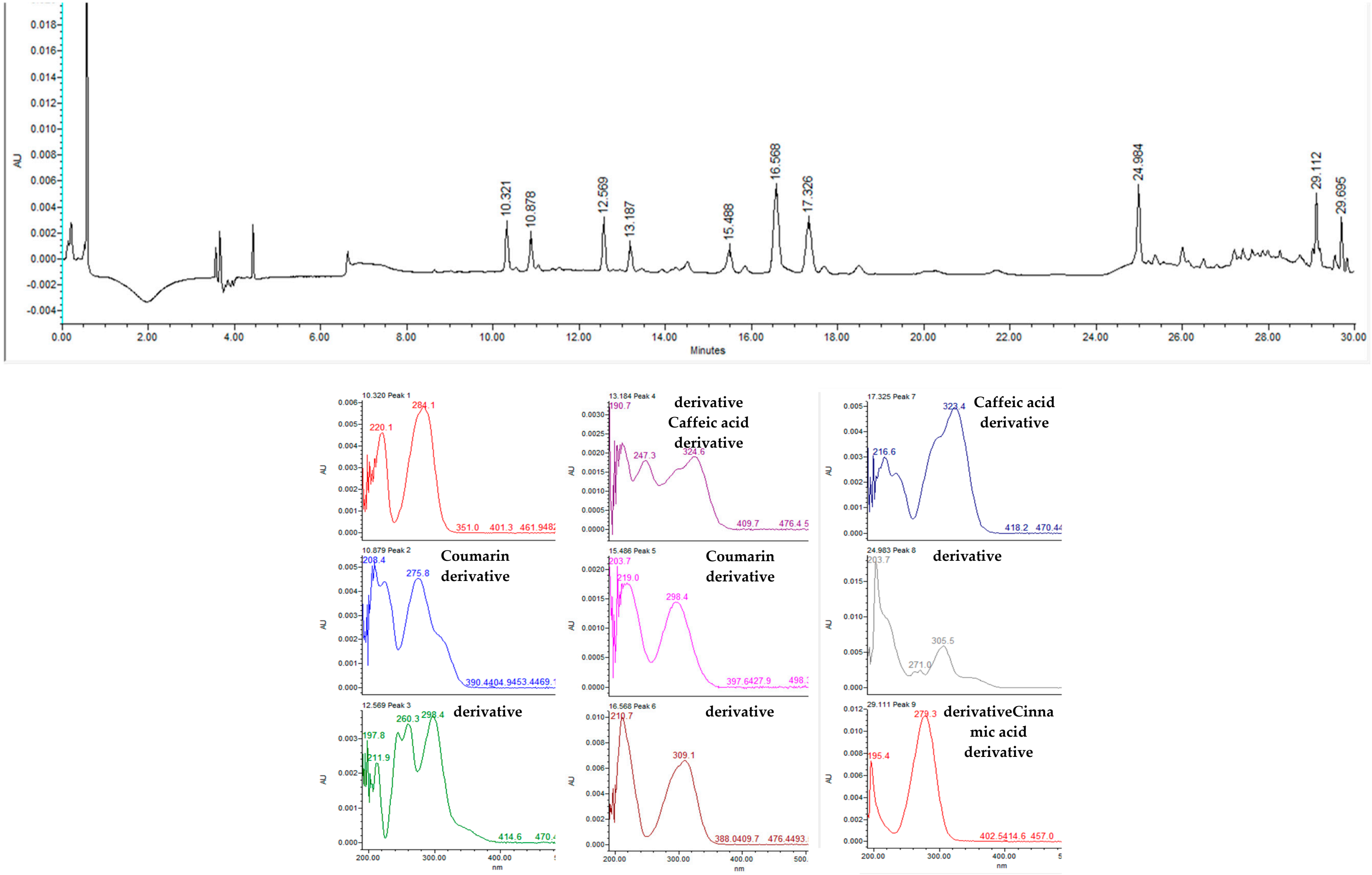


**Figure S4.** Chromatogram and UV absorption spectra of the *Tithonia diversifolia* TdR4 subfraction obtained by HPLC-PDA at $\lambda$=330 nm

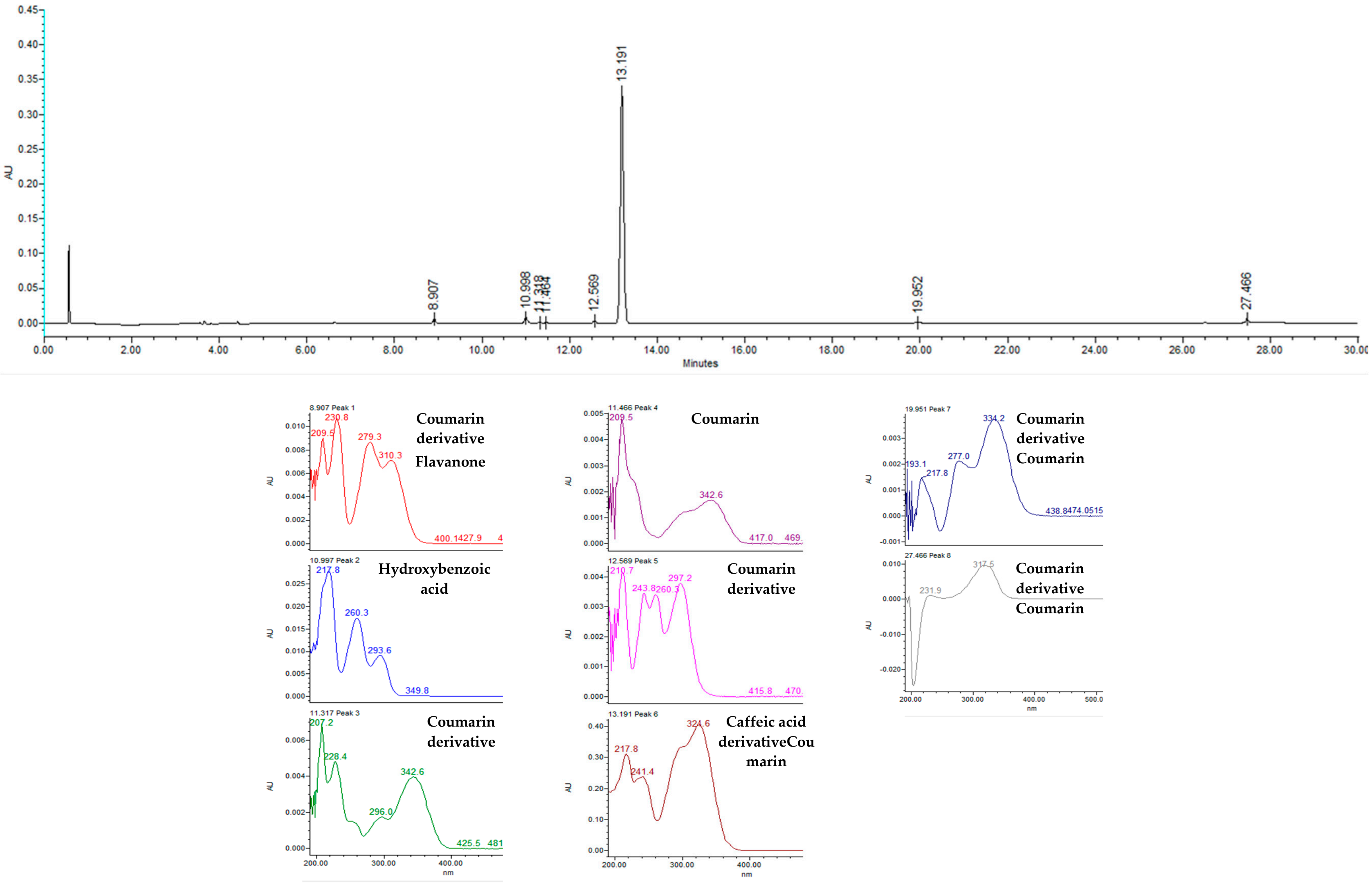


**Figure S5.** Chromatogram and UV absorption spectra of the *Tithonia diversifolia* TdR5 subfraction obtained by HPLC-PDA at $\lambda$=330 nm

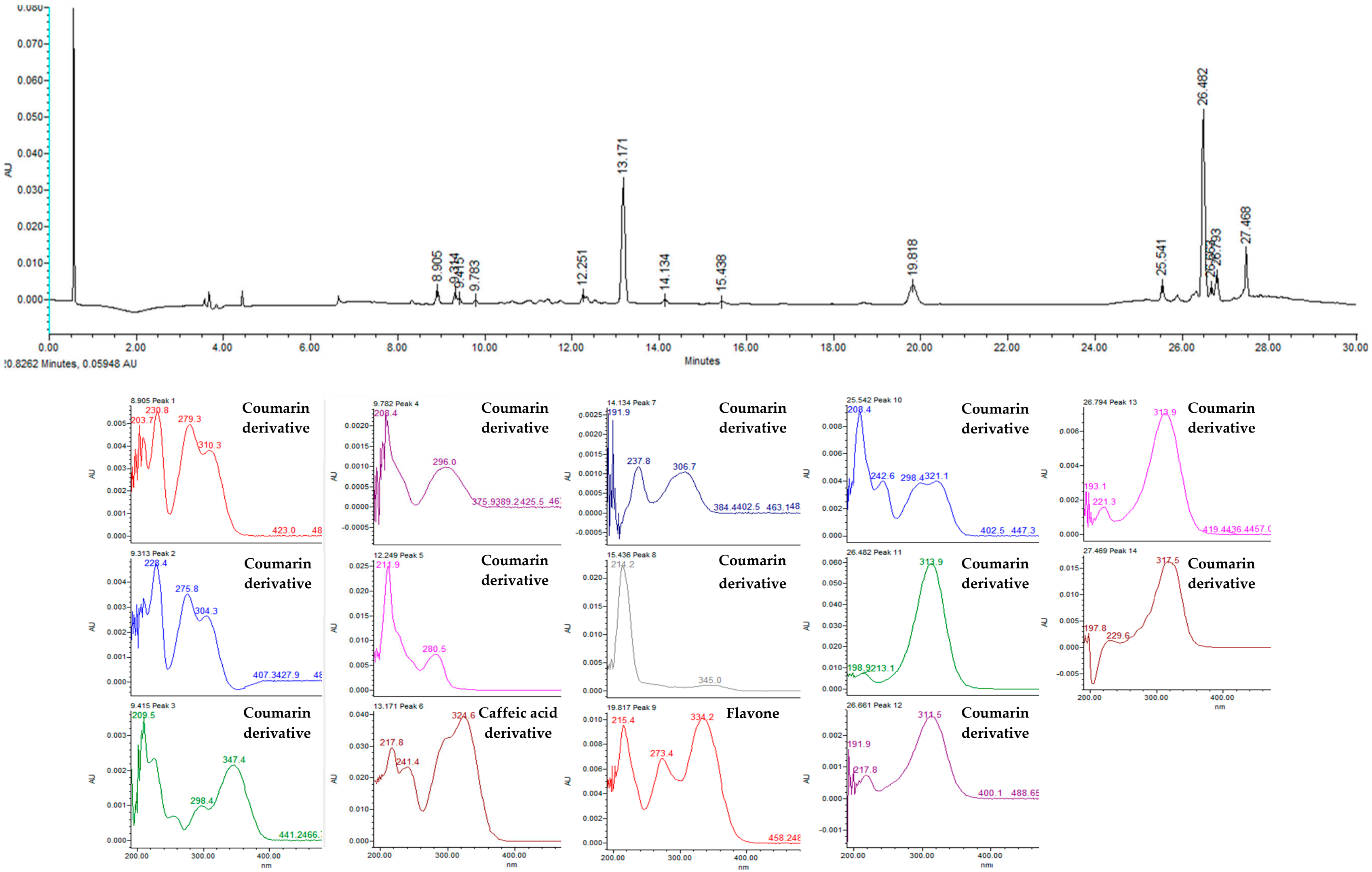


**Figure S6.** Chromatogram and UV absorption spectra of the *Tithonia diversifolia* TdR6 subfraction obtained by HPLC-PDA at λ=330 nm

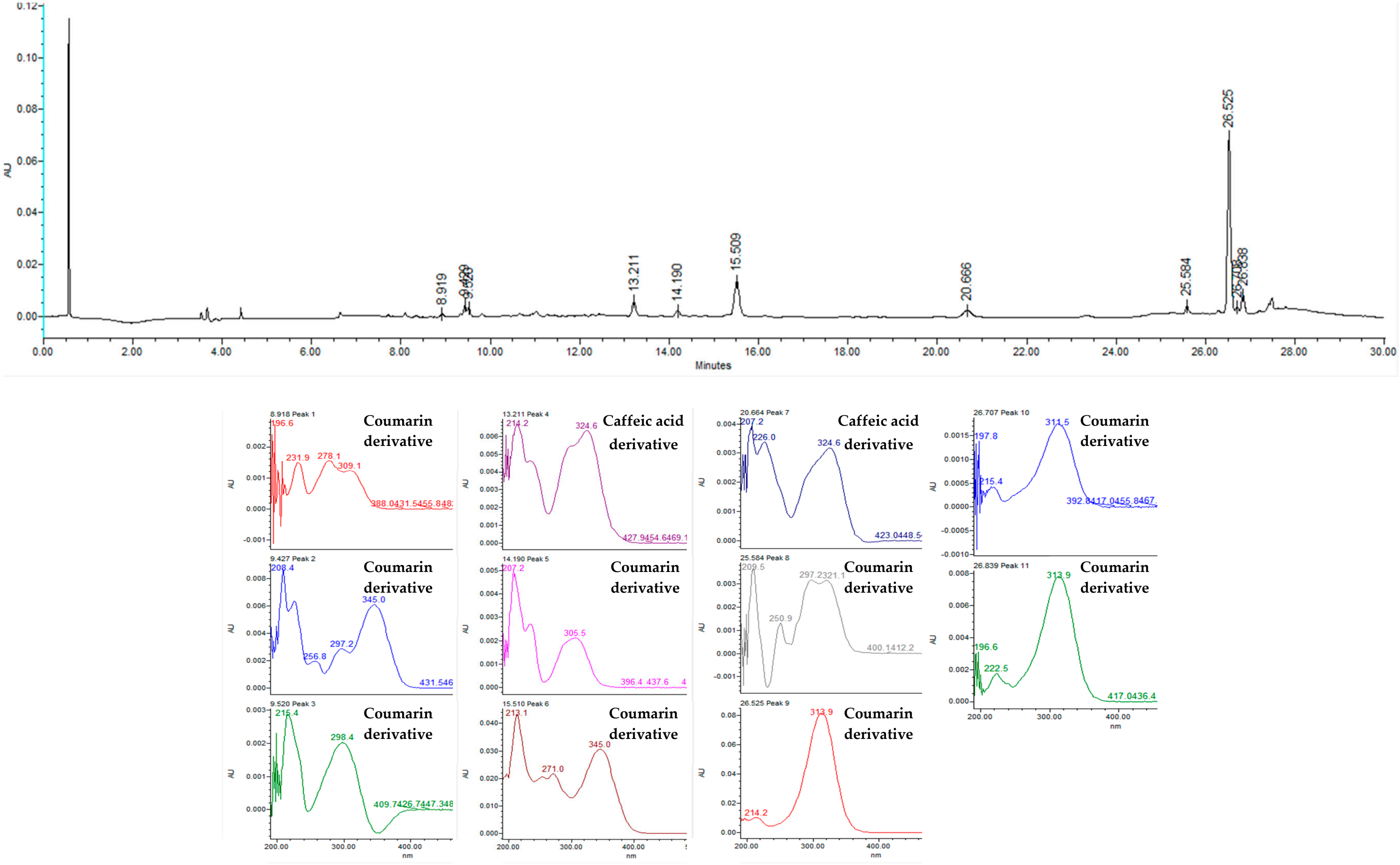


**Figure S7.** Chromatogram and UV absorption spectra of the *Tithonia diversifolia* TdR7 subfraction obtained by HPLC-PDA at $\lambda$=330 nm

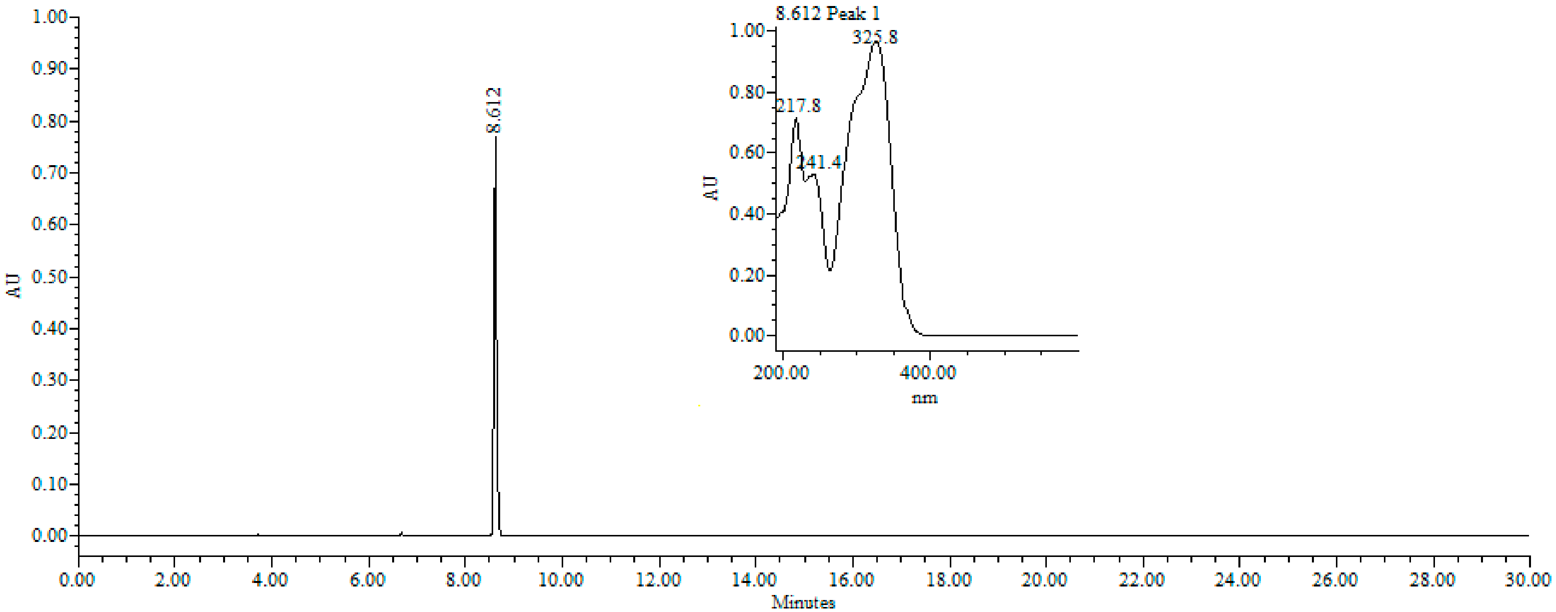


**Figure S8.** Chromatogram and UV absorption spectra of the commercial standard chlorogenic acid, $\lambda$=330 nm

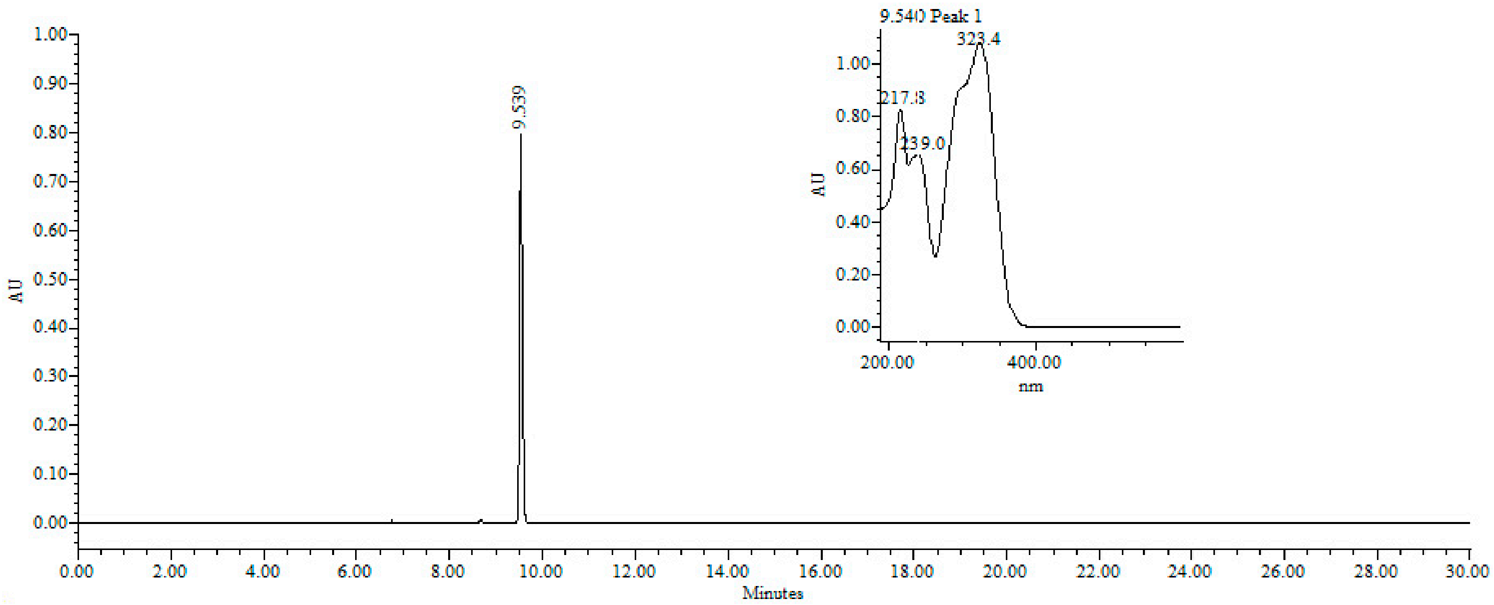


**Figure S9.** Chromatogram and UV absorption spectra of the commercial standard caffeic acid, $\lambda$=330 nm
